# Dioxin Congener Patterns in Breast Milk Samples from Areas Sprayed with Herbicide during the Vietnam War 40 Years after the War Ended

**DOI:** 10.3390/toxics10060323

**Published:** 2022-06-13

**Authors:** Muneko Nishijo, Hoa Thi Vu, Tai Pham-The, Thao Ngoc Pham, Nghi Ngoc Tran, Hideaki Nakagawa, Hisao Nishijo

**Affiliations:** 1 Department of Public Health, Kanazawa Medical University, Uchinada 920-0293, Japan; vuhoa5593hvqy@gmail.com (H.T.V.); phamngocthaovmmu@gmail.com (T.N.P.); 2 Biomedical and Pharmaceutical Research Centre, Vietnam Military Medical University, Hanoi 12108, Vietnam; taithuy@kanazawa-med.ac.jp; 3 Ministry of Health, Vietnam Government, Hanoi 10060, Vietnam; nghi_tranngoc@yahoo.com; 4 Department of Social and Environmental Medicine, Kanazawa Medical University, Uchinada 920-0293, Japan; hnakagaw@kanazawa-med.ac.jp; 5 System Emotional Science, University of Toyama, Sugitani, Toyama 930-0194, Japan; nishijo@med.u-toyama.ac.jp

**Keywords:** dioxin, congener profile, breast milk, herbicide, Vietnam

## Abstract

Large amounts of herbicides containing polychlorinated dibenzo-p-dioxins and dibenzo-furans (PCDD/Fs) were sprayed in South Vietnam during the Vietnam War. Levels of PCDD/Fs in the environment of South Vietnam remained high even 40 years later. A total of 861 breast milk samples (597 from three areas sprayed with herbicides, Quang Tri, Da Nang, and Bien Hoa, and 264 from three unsprayed areas in North Vietnam) were collected between 2007 and 2015 and the PCDD/F concentrations in the samples were determined. Levels of TEQ-PCDD/Fs and 17 PCDD/F congeners were higher in the sprayed area samples than the unsprayed area samples. We found particular PCDD/F congener patterns for different areas. High tetrachlorodibenzo-p-dioxin (TCDD) concentrations were found in Bien Hoa, high TCDD and 1,2,3,6,7,8-hexadibenzo-p-dioxin concentrations were found in Da Nang, and high 1,2,3,4,6,7,8-heptadibenzo-p-dioxin concentrations were found in Quan Tri. High 1,2,3,4,7,8-hexadibenzofuran and 1,2,3,4,6,7,8-heptadibenzofuran concentrations were also found in Da Nang and Quang Tri. However, breast feeding may have caused associations between the TCDD and polychlorinated dibenzofuran congener concentrations. Advanced statistical analysis will need to be performed in future to assess the characteristic PCDD/F congener profiles in breast milk samples from areas of Vietnam previously sprayed with herbicides.

## 1. Introduction

Between 1961 and 1971, during operations by the United States (US) Armed Forces in the Vietnam War, >71 × 10^6^ L of herbicides were sprayed in Vietnam south of the demilitarized zone (the 17th parallel) [1]. Agent Orange, which contains large quantities of 2,3,7,8-tetrachlorodibenzo-p-dioxin (TCDD), the most toxic polychlorinated dibenzo-p-dioxin and dibenzofuran (PCDD/F) congener, comprised approximately two-thirds of the herbicides that were sprayed [2]. Several decades after herbicide spraying ceased, PCDD/F, particularly TCDD, concentrations in environmental media and human tissues remain higher in areas near former US airbases in Da Nang and Bien Hoa than in unsprayed areas in Vietnam [3,4,5].

Historical material provided by the US Department of Defense to the Vietnam Government National Steering Committee on Overcoming the Post-war Consequences of Toxic Chemicals indicate that 150,000 m^3^ of herbicide containing 365,000 ppt of PCDD/Fs were aerially sprayed near the Da Nang airbase between 1965 and 1971 and that 515,000 m^3^ of herbicide containing 962,560 ppt of PCDD/Fs were aerially sprayed near the Bien Hoa airbase between 1965 and 1971. These former US airbases in Vietnam are called PCDD/F contamination hot spots [6]. Hatfield Consultants and the Vietnam Government found extremely high TCDD concentrations in soil and sediment near the Da Nang and Bien Hoa airbases in 2007 and 2010, nearly 40 years after herbicide spraying ended [7,8].

The PCDD/F congeners, including TCDD, are persistent in the environment and resistant to metabolism. PCDD/Fs are lipophilic, so can accumulate in fatty tissues in biota. PCDD/F concentrations in human tissues can increase with age because the biological half-lives of PCDD/F congeners are long (7–11 years for TCDD) [9,10,11]. Breast milk contains fat and can therefore contain PCDD/Fs at high concentrations. Milk samples are more readily collected than blood samples from humans. Therefore, milk samples are often collected to allow human exposure to PCDD/Fs to be monitored and to provide data for epidemiological studies of the toxic effects of PCDD/Fs in humans (e.g., cancer, childhood neurodevelopment, diabetes, and endocrine disruption).

We collected 241 breast milk samples from nursing mothers in the Da Nang cohort recruited in 2008–2009, one month after delivery and found that toxic equivalent (TEQ)-PCDD/Fs were four-times higher than that in breast milk from nursing mothers in unsprayed areas. However, TCDD contributed only ~10% of the TEQ-PCDD/Fs in the breast milk samples from the Da Nang cohort even though the TCDD contributions were higher in the breast milk samples from the unsprayed areas [4]. The mean TEQ-PCDD/Fs levels for 210 breast milk samples collected from another dioxin hot spot near Bien Hoa airbase in 2012 was three-times higher than the mean level for breast milk samples from Hanoi, an unsprayed area, but lower than the mean level for breast milk from Da Nang [5]. However, the TCDD concentrations were higher in breast milk from Bien Hoa than in breast milk from Da Nang. TCDD contributed >25% of the TEQ-PCDD/Fs in the samples from Bien Hoa [5]. These results suggest that dioxin contamination of breast milk has different characteristics in Bien Hoa and Da Nang. We also found higher concentrations of PCDD/F congeners other than TCDD in breast milk from Da Nang [4] and Bien Hoa [5] than in breast milk from unsprayed areas. However, we have not previously assessed differences between the concentrations of PCDD/F congeners other than TCDD in samples from Da Nang and Bien Hoa.

In 2002–2003, we determined the concentrations of PCDD/F congeners in breast milk samples from mothers living in Quang Tri, which was heavily sprayed with herbicides during the Vietnam War but is located far from former US airbases. We found higher concentrations of high-chlorinated PCDD/F congeners such as hexachlorodibenzo-p-dioxins (HexaCDD) and heptachlorodibenzo-p-dioxin (HeptaCDD) in the samples from Quang Tri [12] than were found in breast milk samples collected in the 1970s and 1980s from Da Nang and Dong Nai (where the Bien Hoa airbase is located) by Schecter et al. [13] and Dwernychuk et al. [14]. These findings suggest that the PCDD/F congener patterns in the breast milk samples collected soon after Vietnam War might be different from the patterns in the breast milk samples collected almost 40 years after the war ended.

Therefore, we investigated PCDD/F congener patterns in breast milk samples from dioxin contamination hot spots nearby former US military airbases (Da Nang and Bien Hoa), a sprayed area only in the past time (Quang Tri), and unsprayed areas to clarify the characteristics of dioxin contamination in areas of Vietnam that have been sprayed with herbicides 40 years after the Vietnam War ended.

## 2. Materials and Methods

### 2.1. Study Areas and Subjects

**Figure 1:**
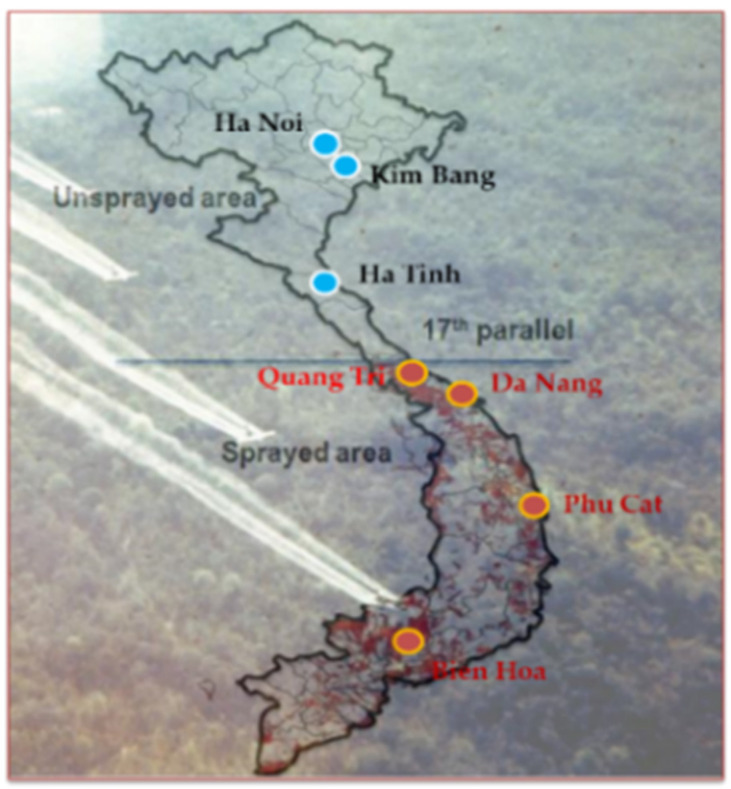
A map of Vietnam.

Samples from the dioxin contamination hot spots around Da Nang and Bien Hoa airbases were analyzed. Samples from 227 mothers living in Thanh Khe and Son Tra districts in Da Nang city and <10 km from the Da Nang airbase who participated in a birth cohort study in 2008 and 2009 [15] were analyzed. Samples from 283 mothers living in 10 communes around Bien Hoa airbase (another hot spot) in Bien Hoa city and who gave birth at Dong Nai General Hospital were recruited to birth cohort studies in 2012 and 2015 [16] were analyzed. The locations of these hot spots are shown on a map of Vietnam in Figure 1.

Samples from 87 mothers living in a sprayed area in Cam Chinh commune in Cam Lo district, Quang Tri province, who had been recruited to surveys in 2002 and 2003 [12,17] were analyzed. Quang Tri province is ~30 km south of the 17th parallel, the demilitarized zone (see Figure 1), and was heavily sprayed during the Vietnam War.

Samples from the unsprayed areas marked blue in Figure 1 were also analyzed. Samples from 62 mothers in the Cam Phuc commune in Cam Xuyen district, Ha Tinh province, ~280 km north of the 17th parallel enrolled in a survey in 2003 were analyzed. Samples from 75 mothers in Kim Bang district, Ha Nam province, ~50 km from the center of Hanoi city in northern Vietnam, enrolled in a survey in 2008 [15,18,19] were analyzed. Samples from 127 mothers in Ha Dong district in Hanoi city enrolled in a birth cohort study in 2014 [16] were also analyzed. None of the unsprayed areas had been affected by industrial pollution and were treated as areas with only background dioxin contamination. 

In total, 861 breast milk samples were analyzed. A total of 597 of the samples were from mothers in three dioxin contaminated areas, and the other 264 samples were from mothers in three areas with background levels of dioxins.

Written informed consent was obtained from all mothers in both areas according to a process reviewed and approved by the Health Department of Da Nang City and Dong Nai Province in Vietnam, Hanoi Medical University, and Vietnam Military Medical University. The institutional ethics board at Kanazawa Medical University (Japan) approved the design of this study (No. ES-187).

### 2.2. Determining Dioxin Concentrations in Breast Milk

We collected ~20 mL of breast milk from each mother at her home with assistance from community health station medical staff 1–6 months after the mother had given birth. Each milk sample was frozen and then transferred packed with dry ice to Kanazawa Medical University in Japan. The samples were then stored at −30 °C until the concentrations of 2,3,7,8-chlorinated PCDD/F congeners in the samples were determined.

Milk fat was extracted from each sample and the milk fat content of the sample was determined gravimetrically, then 2,3,7,8-chlorinated ^13^C-labeled PCDD/F internal standards (DF-LCS-A40; Wellington Laboratories, Guelph, Canada) were added. The milk fat was then purified and fractionated using previously published methods [20], then the extract was analyzed using an HP-6980 gas chromatograph (Agilent Technologies, Santa Clara, CA, USA) equipped with an MStation-JMS700 high-resolution mass spectrometer (JEOL, Tokyo, Japan) to determine the concentrations of 17 PCDD and PCDF congeners. The methods used to determine the PCDD/F concentrations in the milk samples were described in previous publications [4,5,21]. For quality control, a certificated reference material of natural milk powder (CRM 607, Community Bureau of reference-BCR, European Commission, Belgium) diluted was analyzed after dilution in distilled water. Recovery rates of ^13^C-labeled internal standards for every individual PCDD/F congener were within 50–115%. Limit of detection (LOD) was defined as a signal-to-noise (S/N) ratio of peak height of chromatogram = 3. LODs for each congener of our measurement are shown in Table 1. The values of the concentrations of congeners below LODs were set to half of the detection limits for statistical analysis.

The TEQ value for each PCDD/F congener in a sample was calculated by multiplying the concentration of the congener by the toxic equivalence factor for the congener published by the World Health Organization in 2005 [22], and then the TEQ-PCDD/Fs level for the sample was calculated by adding together the TEQ values for all of the congeners. 

### 2.3. Statistical Analysis

Statistical analyses were performed using SPSS version 22.0 software (IBM, Armonk, NY, USA). 

The levels of 17 PCDD/F congeners and the TEQ-PCDD/F in the breast milk samples were logarithmically (base 10) transformed to make the distributions match normal distributions more closely, and then the levels in the samples from Quang Tri, Da Nang, Bien Hoa, and the unsprayed areas were compared using general linear models after adjusting the data for maternal age (in years) and parity (primiparae and multiparae).

The concentrations of each PCDD/F congener in the samples from the three dioxin contaminated areas were stratified into low, middle, and high concentrations using the 95th percentile concentration for the samples from the unsprayed areas as the cut-off for the low and middle concentrations. The cut-off for the middle and high concentrations was the 75th percentile concentration for the Da Nang samples. The odds ratios (ORs) for high concentrations of four PCDD/F congeners in the samples from Da Nang and Bien Hoa were compared with the ORs for Quang Tri using a binary logistic regression model after adjusting for maternal age and parity (primiparae and multiparae).

Associations between the TCDD concentrations and the concentrations of four PCDD/F congeners were identified by a linear regression model for the different areas after adjusting for maternal age and parity for all mothers or adjusting for maternal age for primiparae or multiparae mothers.

For all tests, *p* < 0.05 was taken to indicate statistical significance.

## 3. Results

The mean concentrations of 17 PCDD/F congeners and TEQ-PCDD/F levels in the breast milk samples from mothers living in Quang Tri (an area in which herbicide was sprayed during the Vietnam War), the dioxin hot spots in Da Nang and Bien Hoa, and the unsprayed areas were compared after adjusting for age and parity, and the results are shown in Table 1. The TEQ-PCDD/F levels were significantly higher in the Quang Tri, Da Nang, and Bien Hoa samples than the unsprayed area samples. The concentrations of all of the PCDD/F congeners were significantly higher in the Bien Hoa samples than the unsprayed area samples. The concentrations of most of the PCDD/F congeners were significantly higher in the Da Nang and Quang Tri samples than the unsprayed area samples. However, the 1,2,3,7,8-pentachlorodibenzofuran (PentaCDF) and 2,3,4,6,7,8-hexachlorodibenzofuran (HexaCDF) concentrations were not significantly higher in the Quang Tri samples than the unsprayed area samples and the 1,2,3,7,8,9-HexaCDF concentrations were not significantly higher in the Da Nang samples than the unsprayed area samples.

The median TEQ-PCDD/F levels were more than two- or three-times higher in the Quang Tri, Da Nang, and Bien Hoa samples than in the unsprayed area samples. The median TCDD concentrations were three-times higher in the Da Nang and Bien Hoa samples than the unsprayed area samples, but the median TCDD concentration was only 1.3-times higher in the Quang Tri samples than the unsprayed area samples. The median concentrations of the other PCDD congeners were higher in the samples from the three exposed areas than the unsprayed areas. In particular, the median 1,2,3,6,7,8-HexaCDD and 1,2,3,4,6,7,8-HeptaCDD concentrations were higher in the Quang Tri and Da Nang samples than the Bien Hoa samples, as shown in Table 1. The median 1,2,3,4,7,8-HexaCDF, 1,2,3,6,7,8-HexaCDF, and 1,2,3,4,6,7,8-HeptaCDF concentrations were higher in the samples from the three exposed areas than the unsprayed areas, and the concentrations were two-times higher in the Quang Tri and Da Nang samples than the Bien Hoa samples, as shown in Table 1. 

**Figure 2:**
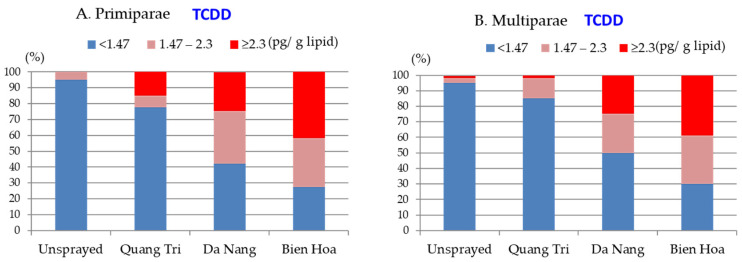
Percentages of samples from primiparae (**A**) and multiparae (**B**) mothers living in Quang Tri, Da Nang, Bien Hoa, and unsprayed areas containing TCDD at low, middle, and high concentrations.

**Figure 3:**
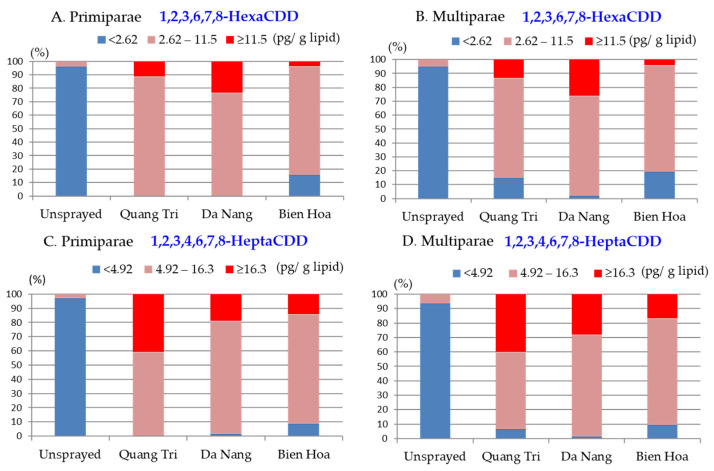
Percentages of samples from (**A**,**C**) primiparae and (**B**,**D**) multiparae living in Quang Tri, Da Nang, Bien Hoa, and unsprayed areas containing 1,2,3,6,7,8-HexaCDD (**A**,**B**) and 1,2,3,4,6,7,8-HeptaCDD (**C**,**D**) at low, middle, and high concentrations.

**Figure 4:**
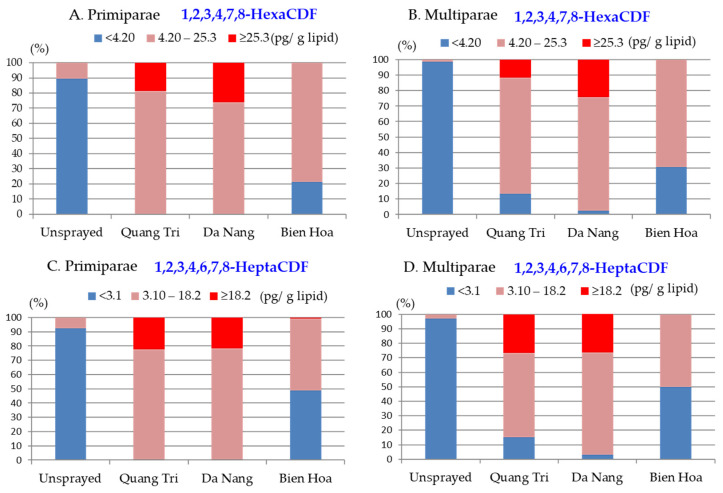
Percentages of samples from (**A**,**C**) primiparae and (**B**,**D**) multiparae living in Quang Tri, Da Nang, Bien Hoa, and unsprayed areas containing levels of 1,2,3,4,7,8-HexaCDF (**A**,**B**) and 1,2,3,4,6,7,8-HeptaCDF (**C**,**D**) at low, middle, and high concentrations.

After assessing these median concentrations, we selected five congeners (TCDD, 1,2,3,6,7,8-HexaCDD, 1,2,3,4,6,7,8-HeptaCDD, 1,2,3,4,7,8-HexaCDF, and 1,2,3,4,6,7,8-HeptaCDF) and calculated the percentages of the samples from primiparae and multiparae mothers living in Quang Tri, Da Nang, Bien Hoa, and unsprayed areas that contained these congeners at low, middle, and high concentrations. The results are shown in Figure 2, Figure 3 and Figure 4. The TCDD concentrations were high (≥2.3 pg/(g lipid)) in ~15%, ~25%, ~40%, and none of the samples from primipara mothers living in Quang Tri, Da Nang, Bien Hoa, and the unsprayed areas, respectively, as shown in Figure 2A. High TCDD concentrations were found in similar percentages of samples from multipara and primipara mothers living in Da Nang and Bien Hoa but fewer samples from multipara mothers (<5%) than primipara mothers living in Quang Tri, as shown in Figure 2B.

Areas with statistically different percentages of samples containing high TCDD concentrations were identified using a binary logistic regression model after adjusting for maternal age and primipara and multipara mothers, and the results are shown in Table 2. For primipara mothers, the OR for a high TCDD concentration was significantly higher for Bien Hoa (OR 3.7) than the other areas, indicating that the risks of the TCDD concentration being high were similar for Da Nang and Quan Tri but significantly higher for Bien Hoa than Quang Tri. For multipara mothers, the ORs were high for Da Nang and Bien Hoa (ORs 19.5 and 36.6, respectively) relative to Quang Tri, indicating that the risks of the TCDD concentration being high were higher for Da Nang and Bien Hoa than Quan Tri. For all mothers, the OR for a high TCDD concentration after adjusting for age and parity was almost twice as high for Bien Hoa (OR 10.0) as for Da Nang (OR 5.6), indicating that the risk of the TCDD concentration being high was higher for Bien Hoa than the other herbicide-sprayed areas.

The 1,2,3,6,7,8-HexaCDD concentrations were high (≥11.5 pg/g lipid) in ~10%, ~25%, and <5% of the samples from primipara mothers living in Quang Tri, Da Nang, and Bien Hoa, respectively, and middle 1,2,3,6,7,8-HexaCDD concentrations (2.6–11.5 pg/g lipid) were found in 85% of the samples from primipara mothers living in Bien Hoa, as shown in Figure 3A. The 1,2,3,6,7,8-HexaCDD concentrations were similar for samples from multipara and primipara mothers, although the percentages of samples with low concentrations were slightly higher for samples from multiparae than primiparae living in Quang Tri and Bien Hoa, as shown in Figure 3B. The 1,2,3,4,6,7,8-HeptaCDD concentrations were high (≥16.3 pg/g lipid) in 40%, 20–25%, and 15% of the samples from primipara and multipara mothers living in Quang Tri, Da Nang, and Bien Hoa, respectively, as shown in Figure 3C,D.

The ORs for high 1,2,3,6,7,8-HexaCDD concentrations in the Da Nang, and Bien Hoa samples relative to the Quang Tri samples are shown in Table 3. The ORs were similar for samples from primipara and multipara mothers living in Da Nang (2.6 and 2.2, respectively) and Bien Hoa (0.25 and 0.25, respectively), indicating that the risks were higher in Da Nang and lower in Bien Hoa than in Quang Tri. For both primipara and multipara mothers, the risks of high 1,2,3,6,7,8-HexaCDD concentrations were significantly higher in Da Nang and significantly lower in Bien Hoa than in Quang Tri.

The ORs for high 1,2,3,4,6,7,8-HeptaCDD concentrations in the Da Nang and Bien Hoa samples were low relative to the ORs for the Quan Tri samples (OR 1.0) for both primipara and multipara mothers, but the ORs were significantly lower only for the Bien Hoa samples. However, for both parity groups, the ORs for high 1,2,3,4,6,7,8-HeptaCDD concentrations were significantly lower for both the Da Nang and Bien Hoa samples than the Quang Tri samples, indicating that the risks of high 1,2,3,4,6,7,8-HeptaCDD concentrations were lower in Da Nang and Bien Hoa than Quang Tri, as shown in Table 3.

The 1,2,3,4,7,8-HexaCDF concentrations were high (≥25.3 pg/g lipid) in 10–20% of the Quang Tri samples and 25% of the Da Nang samples from primipara mothers (Figure 4A) and multipara mothers (Figure 4B), but none of the Bien Hoa samples had high concentrations. The 1,2,3,4,6,7,8-HeptaCDF concentrations were high (≥18.2 pg/g lipid) in similar percentages of the Quang Tri and Da Nang samples from primipara mothers (20%; Figure 4C) and multipara mothers (25%; Figure 4D). Very few of the Bien Hoa samples exhibited high 1,2,3,4,6,7,8-HeptaCDF concentrations, and around half of the samples from primipara and multipara mothers had middle concentrations, as shown in Figure 4C,D).

No samples from Bien Hoa contained high 1,2,3,4,7,8-HexaCDF and 1,2,3,4,6,7,8-HeptaCDF concentrations, so ORs were calculated only for Da Nang relative to Quang Tri (data not shown). However, the ORs for high 1,2,3,4,7,8-HexaCDF and 1,2,3,4,6,7,8-HeptaCDF concentrations were not significantly different for Da Nang (0.98 and 1.03, respectively) than Quang Tri, indicating similar risks of exposure to high 1,2,3,4,7,8-HexaCDF and 1,2,3,4,6,7,8-HeptaCDF concentrations in Da Nang and Quang Tri.

Associations between the TCDD concentrations and the concentrations of other PCDD/F congeners found at high concentrations in the sprayed areas (1,2,3,6,7,8-HexaCDD, 1,2,3,4,6,7,8-HeptaCDD, 1,2,3,4,7,8-HxCDF, and 1,2,3,4,6,7,8-HeptaCDF) were assessed after adjusting for maternal age and parity. Standardized β values for each area are shown in Table 4. Significant associations were found between the TCDD concentrations and the 1,2,3,6,7,8-HexaCDD, 1,2,3,4,6,7,8-HeptaCDD, 1,2,3,4,7,8-HxCDF, and 1,2,3,4,6,7,8-HeptaCDF concentrations for all of the areas, including the unsprayed areas. However, the highest β values for 1,2,3,6,7,8-HexaCDD, 1,2,3,4,6,7,8-HeptaCDD, and 1,2,3,4,7,8-HxCDF (i.e., not 1,2,3,4,6,7,8-HeptaCDF) were for Quang Tri, followed by Da Nang and Bien Hoa. In particular, the β values for all of the congeners were lower for Bien Hoa than the unsprayed areas and other sprayed areas. The β values for 1,2,3,4,6,7,8-HeptaCDF were lower for all of the sprayed areas than the unsprayed areas, indicating that the 1,2,3,4,6,7,8-HeptaCDF concentration was more weakly associated with the TCDD concentration in Quang Tri, Da Nang, and Bien Hoa than the areas at background concentrations.

The PCDD/F concentrations were lower in the samples from multiparae than primiparae, so associations between the TCDD concentrations and 1,2,3,6,7,8-HexaCDD, 1,2,3,4,6,7,8-HeptaCDD, 1,2,3,4,7,8-HxCDF, and 1,2,3,4,6,7,8-HeptaCDF concentrations in each area were assessed after adjusting for maternal age for primipara and multipara mothers. The results are shown in Table 5. The β values for all of the congeners for unsprayed areas were significant and were higher for primiparae than multiparae. However, the β value for 1,2,3,4,6,7,8-HeptaCDF for primipara mothers in Quang Tri was not significant and was lower than the β value for multipara mothers. The β values for 1,2,3,6,7,8-HexaCDD in Da Nang and 1,2,3,4,6,7,8-HeptaCDD in Bien Hoa were significant but lower for primiparae than multiparae. The β values for 1,2,3,4,7,8-HxCDF and 1,2,3,4,6,7,8-HeptaCDF for primipara mothers in Da Nang and Bien Hoa were not significant but the β values for multipara mothers were significant. These results indicated that TCDD concentrations and 1,2,3,6,7,8-HexaCDD, 1,2,3,4,6,7,8-HeptaCDD, 1,2,3,4,7,8-HxCDF, and 1,2,3,4,6,7,8-HeptaCDF concentrations may be associated in different ways for primipara and multipara mothers.

**Figure 5:**
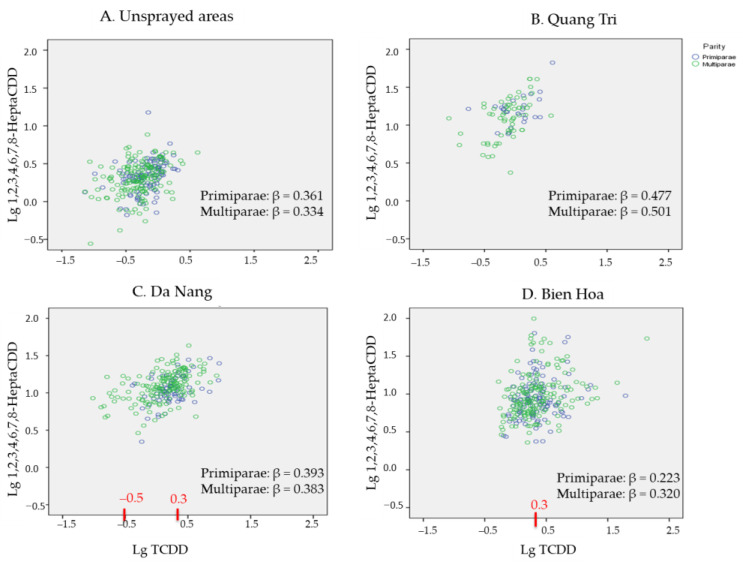
Relationships between the 1,2,3,4,6,7,8-HeptaCDD and TCDD concentrations (shown as base-10 logarithmically transformed values) in the samples from (**A**) unsprayed areas, (**B**) Quang Tri, (**C**) Da Nang, and (**D**) Bien Hoa. Purple circles denote samples from primipara mothers and green circles denote samples from multipara mothers.

**Figure 6:**
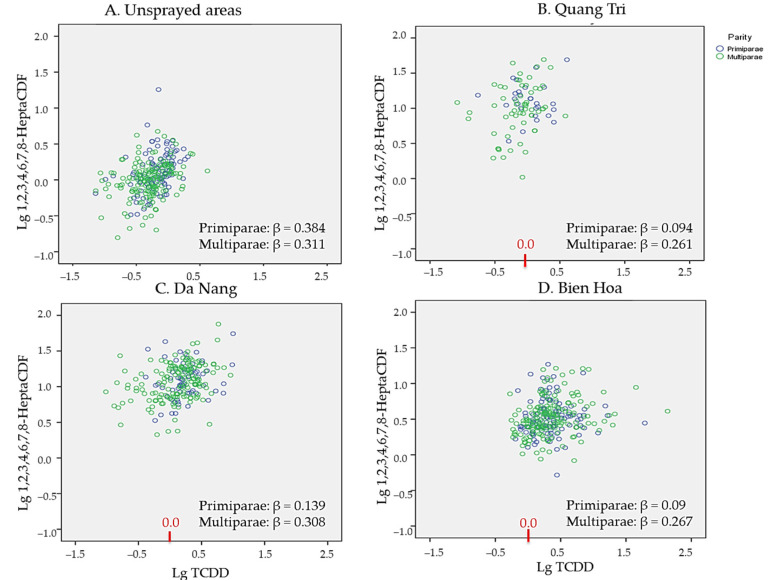
Relationships between the 1,2,3,4,6,7,8-HeptaCDF and TCDD concentrations (shown as base-10 logarithmically transformed values) in the samples from (**A**) the unsprayed areas, (**B**) Quang Tri, (**C**) Da Nang, and (**D**) Bien Hoa. Purple circles denote samples from primipara mothers and green circles denote samples from multipara mothers.

Differences in the associations between the TCDD concentrations and the 1,2,3,6,7,8-HexaCDD, 1,2,3,4,6,7,8-HeptaCDD, 1,2,3,4,7,8-HxCDF, and 1,2,3,4,6,7,8-HeptaCDF concentrations for primipara and multipara mothers were investigated by plotting the 1,2,3,4,6,7,8-HeptaCDD and 1,2,3,4,6,7,8-HeptaCDF concentrations against the TCDD concentrations in the samples from the sprayed and unsprayed areas. The plots are shown in Figure 5 and Figure 6. In each scatter plot, data for the samples from the primipara and multipara mothers are indicated by purple circles and green circles, respectively. The β values for the relationships between the TCDD concentrations and 1,2,3,4,6,7,8-HeptaCDD and 1,2,3,4,6,7,8-HeptaCDF concentrations according to parity are shown in each plot.

The 1,2,3,4,6,7,8-HeptaCDD concentration increased as the TCDD concentration in-creased for both primipara and multipara mothers in Quan Tri as shown in Figure 5B and Da Nang as shown in Figure 5C. However, the 1,2,3,4,6,7,8-HeptaCDD concentrations increased less in samples with high TCDD concentrations (>2.0 pg/g lipid) (0.3 log units) from primipara and multipara mothers from Da Nang than in samples with lower TCDD concentrations, as shown in Figure 5C. This made the association weaker for the samples with TCDD concentrations <0.3 pg/g lipid (−0.5 log units) from multipara mothers because the 1,2,3,4,6,7,8-HeptaCDD concentrations were relatively high. As shown in Figure 5D, some samples with TCDD concentrations >2.0 pg/g lipid (0.3 log units) from Bien Hoa had extremely high 1,2,3,4,6,7,8-HeptaCDD concentrations, meaning the TCDD and 1,2,3,4,6,7,8-HeptaCDD concentrations were weakly associated, particularly for the samples from primipara mothers. 

No significant association was found between the TCDD and 1,2,3,4,6,7,8-HeptaCDF concentrations in the samples from primipara mothers from Quang Tri (β value 0.094; Figure 6B), Da Nang (β value 0.139; Figure 6C), or Bien Hoa (β value 0.09; Figure 6D). However, the samples with TCDD concentrations <1.0 pg/g lipid (log value 0.0) from multipara mothers had relatively low 1,2,3,4,6,7,8-HeptaCDF concentrations, resulting in weak but significant correlations between the TCDD and 1,2,3,4,6,7,8-HeptaCDF concentrations for all of the herbicide-exposed areas (see Figure 6B–D).

The 1,2,3,6,7,8-HexaCDD, 1,2,3,4,6,7,8-HeptaCDD, 1,2,3,4,7,8-HxCDF, and 1,2,3,4,6,7,8-HeptaCDF concentrations in the samples from the herbicide-exposed areas increased as the TCDD concentrations increased, but the strengths of the associations were different for the PCDDs and PCDFs and for samples from primipara and multipara mothers. The 1,2,3,6,7,8-HexaCDD and 1,2,3,4,6,7,8-HeptaCDD concentrations were strongly (more strongly for 1,2,3,6,7,8-HexaCDD than 1,2,3,4,6,7,8-HeptaCDD) associated with the TCDD concentration for the samples from all of the areas and both parity categories, although the association between the TCDD and 1,2,3,4,6,7,8-HeptaCDD concentrations in the samples from primipara mothers from Bien Hoa included samples with TCDD concentrations >2.0 pg/g lipid. No associations were found between the 1,2,3,4,7,8-HxCDF and 1,2,3,4,6,7,8-HeptaCDF concentrations and the TCDD concentrations in the samples from primipara mothers in Da Nang and Bien Hoa. The associations between the TCDD concentration and the 1,2,3,6,7,8-HexaCDD, 1,2,3,4,6,7,8-HeptaCDD, and 1,2,3,4,7,8-HxCDF concentrations were stronger in the samples from Quang Tri than the other areas, including the unsprayed areas, for both parity categories, but the association between the TCDD concentration and the 1,2,3,4,6,7,8-HeptaCDF was not, indicating that the congener profiles for the Quang Tri and Da Nang samples were not the same.

## 4. Discussion

### 4.1. Dioxin Concentrations in Breast Milk from Vietnamese Mothers

The results provide a broad view of the characteristics of PCDD/F congeners in milk from nursing mothers in areas with different levels of dioxin contamination in Vietnam. The TEQ-PCDD/F levels and the concentrations of almost all of the PCDD/F congeners, including TCDD, were significantly higher in the samples from the herbicide-exposed areas (Quang Tri, Da Nang, and Bien Hoa) in South Vietnam than in the samples from the unsprayed areas in North Vietnam. High concentrations of TCDD, 1,2,3,6,7,8-HexaCDD, 1,2,3,4,6,7,8-HeptaCDD, 1,2,3,4,7,8-HexaCDF, and 1,2,3,4,6,7,8-HeptaCDF were specific to herbicide-exposed areas, but characteristic congeners were different for each exposed area.

A high TCDD concentration was characteristic for Bien Hoa for primipara mothers and for Bien Hoa and Da Nang for multipara mothers. A high 1,2,3,6,7,8-HexaCDD concentration was particular for Da Nang but not for Bien Hoa for both parity categories. A high 1,2,3,4,6,7,8-HeptaCDD concentration was particular for Quang Tri. High 1,2,3,4,7,8-HexaCDF and 1,2,3,4,6,7,8-HeptaCDF concentrations were found only in samples from Da Nang and Quang Tri, but not in samples from Bien Hoa. These results indicated that Bien Hoa and Da Nang may be affected by PCDD/Fs in Agent Orange used at air bases but that herbicides other than Agent Orange containing PCDD/Fs with different congener profiles may have been used at Da Nang and at Quang Tri (in the middle of Vietnam).

According to the US Department of Defense, 52,700 containers of Agent Orange, 29,000 containers of Agent White, and 5000 containers of Agent Blue were prepared at Da Nang airbase between 1964 and 1972 and 98,000 containers of Agent Orange (almost twice as much as at Da Nang airbase), 45,000 containers of Agent White, and 16,000 containers of Agent Blue were used at Bien Hoa airbase between 1966 and 1972 [23]. This suggests that TCDD would have been the dominant PCDD/F at Bien Hoa. Other herbicides such as Agent White contained less TCDD and more other congeners and were sprayed in the middle of Vietnam by the US Air Force from Da Nang airbase.

There were some differences between the congener profiles in the samples from Quang Tri and Da Nang, and 1,2,3,4,6,7,8-HeptaCDD and 1,2,3,4,6,7,8-HeptaCDD concentrations were higher and more associated with the TCDD concentration for the Quang Tri samples than the Da Nang samples. These results suggested that PCDD/F exposure may be different in Quang Tri and Da Nang. In Quang Tri (which did not have a nearby airbase), PCDD/F contamination was probably caused by herbicide spraying during the Vietnam War. This would have caused TCDD concentrations to be lower than at PCDD/F contamination hot spots.

In the present study, it was found that strength of associations with increasing TCDD was different between primiparae and multiparae. Two PCDD congeners showed strong associations with TCDD in all areas and in both parity categories, except association between TCDD and 1,2,3,4,6,7,8-HeptaCDD in primipara mothers in Bien Hoa. For two PCDF congeners, however, associations with TCDD were found only in multipara mothers in Da Nang and Bien Hoa. For 1,2,3,4,6,7,8-HeptaCDF, association with TCDD was also observed in multipara mothers in Quan Tri. Since significant associations with TCDD were observed for all these congeners both in primipara and multipara mothers in unsprayed areas, decreased levels of dioxins in maternal body, particularly PCDF congeners with short biological half-life, through breast feeding might be related with significant associations only in multipara mothers. 

The strengths of the associations between the PCDD/F congener concentrations and TCDD concentration were different for samples from primipara and multipara mothers. The 1,2,3,6,7,8-HexaCDD and 1,2,3,4,6,7,8-HeptaCDD concentrations were strongly associated with the TCDD concentrations in the samples from most of the areas and for both parity categories, but no association was found between the TCDD and 1,2,3,4,6,7,8-HeptaCDD concentrations in the samples from primipara mothers from Bien Hoa. Associations between the 1,2,3,4,7,8-HxCDF and 1,2,3,4,6,7,8-HeptaCDF concentrations and the TCDD concentration were found only for the samples from multipara mothers from Da Nang and Bien Hoa. The 1,2,3,4,6,7,8-HeptaCDF concentration was associated with the TCDD concentration for the samples from multipara mothers from Quan Tri. Significant associations were found between the concentrations of these four PCDD/F congeners and the TCDD concentrations in the samples from both primipara and multipara mothers in unsprayed areas, so decreased PCDD/F concentrations (particularly of PCDF congeners, which have shorter biological half-lives than PCDD congeners) in the tissues of mothers through breast feeding may increase associations between the concentrations of these four PCDD/F congeners and the TCDD concentrations. Advanced statistical analysis will be necessary in future to assess effects of breast feeding on the associations among PCDD/F concentrations.

### 4.2. Dioxin Concentrations in Breast Milk from Mothers Exposed to Dioxins in Countries Other Than Vietnam

#### 4.2.1. Dioxin Levels in Breast Milk from Mothers from European Countries

An explosion of a herbicide manufacturing plant in 1976 caused TCDD contamination in Seveso, Italy. Many residents of the surrounding area were exposed to TCDD, and TCDD concentrations of <10–56,000 pg/g were found in serum from people living near the plant [24]. Weiss et al. determined PCDD/F concentrations in breast milk from nursing mothers in Seveso and reference areas in central Milan (urban control) and a rural area in Lombardy in 2000–2001, 25 years after the explosion [25]. The median concentrations of 17 PCDD/F congeners and the TEQ-PCDD/F concentrations that were found in the samples from Seveso, Milan, and Lombardy are shown in Table 6. The TCDD concentrations were more than two-times higher in the samples from Seveso than the samples from Milan and Lombardy, but the 1,2,3,6,7,8-HexaCDD and 1,2,3,4,6,7,8-HeptaCDD concentrations were lower in the samples from Seveso than the samples from Milan and Lombardy. These results indicated that TCDD concentrations were particularly high in the tissues of residents of Seveso even 25 years after the accident.

The Duisburg birth cohort study involved 169 mother–infant pairs recruited at childbirth in the highly industrialized Duisburg area in Germany in 2000–2003 [26]. The PCDD/F concentrations in breast milk samples were determined, and the median concentrations of 17 PCDD/F congeners are shown in Table 6. The congener profiles were similar to the congener profiles for the samples from Milan analyzed in a previous study [24] but the concentrations of the PCDD congeners except TCDD were slightly higher in the Duis-burg samples than the Milan samples. This suggested that industrialization may increase the concentrations of all PCDD/F congeners, particularly 1,2,3,6,7,8-HexaCDD, 1,2,3,4,6,7,8-HeptaCDD, OctaCDD, and 2,3,4,7,8-pentachlorodibenzofuran (PentaCDF), in human tissues. Compared with these congener concentrations in Da Nang and Quang Tri, three PCDD congener concentrations were similar in Duisburg area in Germany. However, the concentrations of 1,2,3,4,7,8-HexaCDF and 1,2,3,4,6,7,8-HeptaCDF were lower in Duisburg. These results suggest high 1,2,3,4,7,8-HexaCDF and 1,2,3,4,6,7,8-HeptaCDF concentrations may be characteristic in Da Nang and Quang Tri in middle Vietnam.

A hazardous waste incinerator was built in Tarragona County, Catalonia, Spain, in 1999. PCDD/F concentrations in breast milk from primipara mothers living near the in-cinerator were determined several times from 2002 onward [27]. The PCDD/F congener profiles in breast milk collected in 2017 (the latest report) are shown in Table 6. The concentrations of all PCDD/F congeners were low, and the median TEQ-PCDD/F was only 2.3 (pg-TEQ/g lipid) [27]. The authors stated that the decreasing PCDD/F levels were associated with decreasing dietary intakes of fatty foods such as fish and meat and that changes in environmental contamination had not affected human exposure to PCDD/Fs owing to the effective management of waste treatment and incineration processes [27].

#### 4.2.2. Dioxin Levels in Breast Milk from Asian Countries

Sun et al. collected milk samples from mothers in three areas in China, Shijiazhuang (an industrial city), Tianjin (a large port city), and Yantai (a coastal city with a strong fishing industry), in 2006–2007 and determined the PCDD/F concentrations in the samples [28]. The median PCDD/F congener and TEQ-PCDD/F levels found in the Tianjin samples are shown in Table 6 because the concentrations were higher than the concentrations in the samples from the other areas. The PCDD/F congener and TEQ-PCDD/F concentrations in the Tianjin samples were generally lower than the concentrations found in samples from Lombardy in Italy in the study mentioned above [24] and similar to the concentrations found in samples from Catalonia in Spain in another study mentioned above [27]. The median levels of PCDD/F congener and TEQ-PCDD/F in unsprayed areas in Vietnam are also similar to levels in the Tianjin samples, suggesting PCDD/Fs contamination in Tianjin in China, Catalonia in Spain, and unsprayed areas in Vietnam may be at background levels.

The concentrations of most PCDD/F congener in breast milk from Hokuriku on the Japanese coast [29] were much higher than the concentrations found in the Tianjin samples [28] and similar to the concentrations found in the Milan samples [24], although the 1,2,3,6,7,8-HexaCDD concentrations were higher and the 1,2,3,4,6,7,8-HeptaCDD concentrations were lower in the Japanese samples than the Tianjin and Milan samples [24].

In 2008, Manh et al. determined the PCDD/F concentrations in breast milk from communities near Phu Cat airbase in Vietnam [18]. Phu Cat airbase, an important US military airbase during the Vietnam War, was used to store 17000 containers of Agent Orange, 9000 containers of Agent White, and 2900 containers of Agent Blue for the Ranch Hand mission [23]. The median PCDD/F congener concentrations in the samples are shown in Table 6. The PCDD/F concentrations were similar to the concentrations we found in the Da Nang samples (high TCDD, 1,2,3,6,7,8-HexaCDD, 1,2,3,4,6,7,8-HeptaCDD, 1,2,3,4,7,8-HexaCDF, and 1,2,3,4,6,7,8-HeptaCDF concentrations). The 1,2,3,7,8-pentachlorodibenzo-p-dioxin (PentaCDD) concentrations were higher than the concentrations found in samples from locations near other airbases in Vietnam, and this caused the TEQ-PCDD/F levels to be high. The PCDD/F congener patterns, particularly the high TCDD, 1,2,3,4,7,8-HexaCDF, and 1,2,3,4,6,7,8-HeptaCDF concentrations, were different from the patterns for samples from other countries, suggesting that PCDD/F exposure caused by herbicide spraying increased not only the TCDD concentrations but also the concentrations of other congeners such as 1,2,3,4,7,8-HexaCDF and 1,2,3,4,6,7,8-HeptaCDF in human tissues.

In Bien Hoa, Vietnam, Schecter et al. [13] reported strikingly high TCDD concentrations ranging from 333–1832 pg/g lipid in breast milk samples collected in 1970 (the last period of the Vietnam War). They also collected milk samples in subsequent years and reported decreasing TCDD concentrations year by year, as 133–266 pg/g lipid in 1973 and 2.1–11 pg/g lipid in 1985–1988 [13]. They did not show concentrations of PCDD/F congeners other than TCDD in breast milk samples, but the concentrations in their breast milk samples can be estimated to be not so high from their concentrations in blood samples collected in the same area [13]. Dwernychuk et al. also determined PCDD/F concentrations in breast milk collected in A So village in Aluoi Valley on central Vietnam in 1999 and reported 1.4–19 pg/g lipid for TCDD [14]. They also showed high total HeptaCDD (3.0–230 pg/g lipid) and total OCDD (28–1800 pg/g lipid), but concentrations of PCDF congeners were generally lower [14]. Because of differences in methodology of PCDD/Fs analysis, comparisons of concentrations between the samples collected before 2000 and in our samples collected after 2000 are not easy to conduct. However, increased concentrations of PCDF congeners observed in our samples might be specific to samples collected 40 years after the cessation of herbicide spraying.

### 4.3. Dioxins in Breast Milk and Other Markers of Exposure to PCDD/Fs

The concentrations of PCDD/Fs in breast milk are often used to indicate exposure of humans to dioxins because the high fat content of breast milk allows reliable measurements to be made. However, PCDD/F concentrations in maternal blood are better for estimating the exposure of a fetus to dioxins during pregnancy, which is associated with adverse health effects of the fetus and infants. The relationships between PCDD/F concentrations in breast milk and maternal blood have therefore been studied.

As part of the Duisburg birth cohort study, Wittsiepe et al. determined the concentrations of PCDD/Fs in blood and breast milk collected simultaneously from some mothers [26]. The median high-chlorinated (hexa-, penta-, and octa-chlorinated) PCDD/F congener concentrations were higher in the blood samples than the breast milk samples, although the low-chlorinated (tetra- and penta-chlorinated) PCDD/F congener concentrations were similar. The concentrations of the high-chlorinated PCDD/F congeners (except for the 1,2,3,4,7,8,9-HeptaCDF concentrations) in the blood and milk samples correlated well (γ = 0.62–0.94) [26].

Correlations between PCDD/F congener concentrations in cord blood and breast milk from mothers in the Bien Hoa birth cohort study in 2012 were determined, and good correlations were found for the low-chlorinated PCDD/F congeners, particularly TCDD, in the cord blood and breast milk samples [30].

The results of the studies described above indicate that PCDD/F concentrations in breast milk reflect maternal exposure to dioxins during pregnancy and are good markers for epidemiological studies of the effects of dioxins on infant health, particularly in Vietnam. TCDD is an important PCDD/F congener and PCDD/F congener analysis is necessary. However, drawing enough blood for dioxin congener analysis from pregnant women is very difficult in Vietnam.

Tolerable daily intake (TDI) of dioxins (1–4 pg-TEQ/kg body weight/day) is determined by the WHO [31] for prevention of adverse health effects with lifetime exposure. During early infancy, breast milk is a main source of postnatal dioxin exposure and estimated daily intake (EDI) of dioxins through breast milk for infants is calculated and compared with TDI values. In the present survey, mean EDI values or Quang Tri, Da Nang, and Bien Hoa were 57.3, 90.3, and 68.4 pg-TEQ/kg body weight/day and significantly higher than that for unsprayed areas (23.5 pg-TEQ/kg body weight/day). Even for unsprayed areas, the EDI value was above the TDI value, which was a similar level compared with countries exposed to dioxins at background levels of PCDD/Fs such as New Zealand [32] and Hungary [33]. When the 95th percentile value of EDI for unsprayed areas is set as cut-off value of high EDI, 60 percent of infants in Quang Tri, 90 percent of infants in Da Nang, and 73 percent of infants in Bien Hoa showed high EDI in the present analysis, suggesting large numbers of infants are at risk for potential adverse health effects due to PCDD/Fs exposure in sprayed areas in Vietnam.

However, no significant difference of mean EDI values between Quang Tri and Bien Hoa was shown, even if there was significant difference of TCDD concentrations originating from herbicides used during the Vietnam War. Therefore, determination of PCDD/F congener concentrations in breast milk samples may be necessary to evaluate PCDD/Fs exposure in the sprayed areas in Vietnam.

## 5. Conclusions

(1) The concentrations of almost all of the PCDD/F congeners were significantly higher in the samples from the herbicide-sprayed areas (Quang Tri, Da Nang, and Bien Hoa) than the unsprayed areas. (2) Different risks of high PCDD/F concentrations were found for the three herbicide-exposed areas. The risk of high TCDD concentrations was high for Bien Hoa, the risks of high TCDD and 1,2,3,6,7,8-HexaCDD concentrations were high for Da Nang, and the risk of high 1,2,3,4,6,7,8-HeptaCDD concentrations was high for Quan Tri. High 1,2,3,4,7,8-HexaCDF and 1,2,3,4,6,7,8-HeptaCDF concentrations were found only in samples from Da Nang and Quang Tri. (3) The associations between the TCDD and 1,2,3,4,7,8-HexaCDF and 1,2,3,4,6,7,8-HeptaCDF concentrations were different for samples from primipara and multipara mothers, suggesting that breast feeding affected PCDF concentrations more than PCDD concentrations. Advanced statistical analysis will be required in future to assess the characteristic PCDD/F congener profiles in breast milk samples from herbicide sprayed areas of Vietnam.

## Figures and Tables

**Table 1:** Adjusted mean concentrations of PCDD/F congeners in breast milk from mothers living in Quang Tri, Da Nang, and Bien Hoa, where herbicides were sprayed during the Vietnam War, and from mothers living in unsprayed areas in North Vietnam.

		Unsprayed areas				Quang Tri					Da Nang					Bien Hoa				
	LOD	*n* = 264				*n* = 87					*n* = 227					*n* = 283				
	(ppt)	Med	GM	GSD	Adj M	Med	GM	GSD	Adj M		Med	GM	GSD	Adj M		Med	GM	GSD	Adj M	
PCDD congeners (pg/g lipid)																				
2,3,7,8-TetraCDD	0.004	0.6	0.6	1.9	0.6	0.8	0.8	2.0	0.8	*	1.4	1.4	2.3	1.4	*	2.0	2.3	2.2	2.1	*
1,2,3,7,8- PentaCDD	0.011	1.3	1.2	1.7	1.3	2.3	2.3	1.9	2.3	*	4.2	4.1	1.6	4.1	*	2.9	2.9	1.6	2.7	*
1,2,3,4,7,8- HexaCDD	0.004	0.6	0.6	1.7	0.6	1.7	1.4	2.4	1.4	*	2.3	2.2	1.6	2.2	*	1.3	1.4	1.6	1.3	*
1,2,3,6,7,8- HexaCDD	0.010	1.4	1.3	1.6	1.3	6.7	6.2	1.8	6.2	*	8.4	8.0	1.7	8.1	*	4.5	4.4	1.8	4.3	*
1,2,3,7,8,9- HexaCDD	0.008	0.6	0.6	1.8	0.6	1.8	1.6	2.2	1.6	*	2.6	2.6	1.7	2.6	*	2.0	2.1	2.0	2.1	*
1,2,3,4,6,7,8- HeptaCDD	0.008	2.2	2.1	1.7	2.1	14.1	13.3	1.8	13.2	*	11.8	11.8	1.6	11.9	*	8.5	9.2	1.9	9.0	*
OctaCDD	0.008	14.7	13.1	2.2	13.4	45.2	44.1	1.8	44	*	64.2	66.7	1.6	67	*	55.2	61.7	1.9	59.8	*
PCDF congeners (pg/g lipid)																				
2,3,7,8-TetraCDF	0.005	0.8	0.8	1.6	0.8	0.5	0.5	1.7	0.5	*	0.5	0.5	2.0	0.5	*	0.6	0.6	1.7	0.6	*
1,2,3,7,8- PentaCDF	0.006	0.6	0.5	1.8	0.5	0.6	0.6	2.1	0.6		1.2	1.2	1.9	1.2	*	0.6	0.6	1.7	0.6	
2,3,4,7,8- PentaCDF	0.010	2.8	2.7	1.5	2.8	4.4	4.3	1.9	4.4	*	7.3	7.0	1.6	7.1	*	3.5	3.6	1.6	3.5	*
1,2,3,4,7,8- HexaCDF	0.008	1.7	1.6	1.8	1.7	14.4	12.6	2.1	12.7	*	17.8	16.9	1.8	17.2	*	5.7	5.8	1.6	5.6	*
1,2,3,6,7,8- HexaCDF	0.003	1.4	1.4	1.6	1.4	8.3	7.4	2.1	7.4	*	11.0	10.3	1.8	10.4	*	3.3	3.4	1.6	3.3	*
1,2,3,7,8,9- HexaCDF	0.004	0.3	0.3	2.1	0.3	1.0	1.0	2.3	1.0	*	0.2	0.2	2.3	0.3		0.4	0.5	1.8	0.5	*
2,3,4,6,7,8- HexaCDF	0.006	0.5	0.3	2.8	0.3	0.3	0.3	2.9	0.3		1.3	1.3	1.7	1.3	*	0.7	0.7	1.7	0.7	*
1,2,3,4,6,7,8- HeptaCDF	0.010	1.1	1.1	1.8	1.1	10.8	10.4	2.2	10.4	*	11.9	11.9	1.8	12.0	*	3.2	3.3	1.8	3.3	*
1,2,3,4,7,8,9- HeptaCDF	0.004	0.2	0.2	2.3	0.2	1.6	1.4	2.8	1.4	*	1.3	1.2	2.2	1.2	*	0.6	0.7	1.9	0.6	*
OctaCDF	0.013	0.5	0.5	3.6	0.5	0.2	0.2	3.3	0.2		0.5	0.6	2.4	0.6	*	1.6	1.6	2.1	1.6	*
TEQs (pg/g lipid)																				
TEQ-PCDD/Fs		3.7	3.7	1.5	3.8	9.7	9.1	1.8	9.1	*	12.8	12.5	1.6	12.7	*	8.5	9.0	1.7	8.6	*

LOD: limit of detection, *n*: number of subjects, Med: median, GM: geometric mean, GSD: geometric standard deviation, Adj M: mean adjusted for age and parity (1: primiparae, 2: multiparae); CDD: chlorodibenzo-p-dioxin, CDF: chlorodibenzofuran, TEQ: toxic equivalent, *: *p* < 0.05 at Bonferroni correction for multiple comparisons compared with unsprayed areas.

**Table 2:** Adjusted odds ratios (ORs) for high TCDD concentrations in breast milk from mothers living in Da Nang and Bien Hoa relative to those in breast milk from mothers living in Quang Tri.

	Quang Tri			Da Nang				Bien Hoa			
	*n*	%	OR	*n*	%	OR		*n*	%	OR	
Primiparae	4	14.8	1.0	17	24.6	2.1		47	41.6	3.7	*
Multiparae	1	1.7	1.0	39	24.7	19.5	**	66	38.8	36.6	***
All	5	5.7	1.0	56	24.7	5.6	***	113	39.9	10.0	***

*n*: number of subjects, OR: odds ratio, *: *p* < 0.05, **: *p* < 0.01, ***: *p* < 0.001 after adjusting for age or age and parity.

**Table 3:** Adjusted odds ratios (ORs) for high 1,2,3,6,7,8-HexaCDD and 1,2,3,4,6,7,8-HeptaCDD concentrations in breast milk from mothers living in Da Nang and Bien Hoa relative to those in breast milk from mothers living in Quang Tri.

	Quang Tri			Da Nang				Bien Hoa			
	*n*	%	OR	*n*	%	OR		*n*	%	OR	
1,2,3,6,7,8-HexaCDD											
Primiparae	3	11.1	1.0	16	23.2	2.6		4	3.5	0.25	
Multiparae	8	13.3	1.0	41	25.9	2.2		7	4.1	0.25	*
All	11	12.6	1.0	57	25.1	2.4	*	11	3.9	0.25	**
1,2,3,4,6,7,8-HeptaCDD											
Primiparae	11	40.7	1.0	13	18.8	0.37		16	14.2	0.20	***
Multiparae	24	40.0	1.0	44	27.8	0.57		28	16.5	0.27	***
All	35	40.2	1.0	57	25.1	0.50	*	44	15.5	0.24	***

*n*: number of subjects, OR: odds ratio, *: *p* < 0.05, **: *p* < 0.01, ***: *p* < 0.001 after adjusting for age or age and parity.

**Table 4:** Adjusted associations between the TCDD concentrations and concentrations of other PCDD/F congeners that were found at high concentrations in the herbicide sprayed areas.

	Unsprayed Areas		Dioxin Exposed Areas					
	(*n* = 264)		Quang Tri (*n* = 87)		Da Nang (*n* = 227)		Bien Hoa (*n* = 283)	
	β	*p*-Value	β	*p*-Value	β	*p*-Value	β	*p*-Value
1,2,3,6,7,8-HexaCDD	0.476	***	0.559	***	0.518	***	0.438	***
1,2,3,4,6,7,8-HeptaCDD	0.351	***	0.501	***	0.385	***	0.285	***
1,2,3,4,7,8-HexaCDF	0.332	***	0.429	***	0.392	***	0.258	***
1,2,3,4,6,7,8-HeptaCDF	0.332	***	0.229	*	0.271	***	0.205	**

*n*: number of subjects, β: standardized beta; CDD: chlorodibenzo-p-dioxin; CDF: chlorodibenzofuran; *: *p* < 0.05, **: *p* < 0.01, ***: *p* < 0.001 after adjusting for age and parity (1: primiparae, 2: multiparae).

**Table 5:** Age-adjusted associations between TCDD concentrations and concentrations of four other PCDD/F congeners according to maternal parity.

	Primiparae								Multiparae							
	Unsprayed Areas		Dioxin Exposed Areas						Unsprayed Areas		Dioxin Exposed Areas					
			Quang Tri		Da Nang		Bien Hoa				Quang Tri		Da Nang		Bien Hoa	
	*n* = 105		*n* = 27		*n* = 69		*n* = 113		*n* = 159		*n* = 60		*n* = 158		*n* = 170	
	β	*p*-Value	β	*p*-Value	β	*p*-Value	β	*p*-Value	β	*p*-Value	β	*p*-Value	β	*p*-Value	β	*p*-Value
1,2,3,6,7,8-HexaCDD	0.586	***	0.590	**	0.303	**	0.448	***	0.415	***	0.579	***	0.574	***	0.437	***
1,2,3,4,6,7,8- HeptaCDD	0.361	***	0.477	***	0.393	**	0.223	*	0.334	***	0.501	***	0.383	***	0.320	***
1,2,3,4,7,8-HexaCDF	0.401	***	0.478	***	0.214		0.170		0.303	***	0.435	**	0.439	***	0.307	***
1,2,3,4,6,7,8- HeptaCDF	0.384	***	0.094		0.139		0.090		0.311	***	0.261	*	0.308	***	0.267	**

β: standardized regression coefficient, *n*: number of subjects; CDD: chlorodibenzo-p-dioxin; CDF: chlorodibenzofuran; *: *p* < 0.05, **: *p* < 0.01, ***: *p* < 0.001 after adjusting for maternal age.

**Table 6:** Median levels of PCDD/F congeners and TEQ-PCDD/Fs in breast milk from various countries.

	Italy [21]			Germany [22]	Spain [23]	China [24]	Japan [25]	Vietnam [14]
	Seveso	Milan	Lombardy	Duisburg	Catalonia	Tianjin	Hokuriku	PhuCat
	*n* = 12	*n* = 12	*n* = 12	*n* = 169	*n* = 20	*n* = 20	*n* = 75	*n* = 16
PCDD congeners (pg/g lipid)								
2,3,7,8-TetraCDD	3.7	1.6	1.4	1.5	0.3	0.8	0.8	2.1
1,2,3,7,8-PentaCDD	2.4	3.6	3.3	4.0	0.8	1.6	4.2	5.7
1,2,3,4,7,8-HexaCDD	1.3	1.8	1.7	2.7	0.5	0.8	1.4	2.5
1,2,3,6,7,8-HexaCDD	7.3	9.5	8.6	11.6	2.6	2.1	13.7	10.0
1,2,3,7,8,9-HexaCDD	1.0	1.4	1.3	2.3	0.5	0.6	1.9	3.4
1,2,3,4,6,7,8-HeptaCDD	7.3	11.5	10.2	12.6	2.8	3.0	6.8	17.1
OctaCDD	38.6	49.5	50.2	70.4	15.7	20.5	56.2	80.9
PCDF congeners (pg/g lipid)								
2,3,7,8-TetraCDF	nd	nd	nd	0.3	0.2	0.7	1.0	0.7
1,2,3,7,8-PentaCDF	nd	0.2	nd	0.2	0.1	0.6	0.3	2.4
2,3,4,7,8-PentaCDF	5.9	8.9	7.3	10.1	1.9	5.6	8.1	7.6
1,2,3,4,7,8-HexaCDF	2.7	3.3	2.8	2.7	0.6	2.9	2.3	18.7
1,2,3,6,7,8-HexaCDF	1.7	2.4	1.9	2.4	0.6	2.2	2.3	10.6
1,2,3,7,8,9-HexaCDF	nd	nd	nd	1.0	0.4	nd	1.5	0.5
2,3,4,6,7,8-HexaCDF	0.6	0.9	0.7	0.1	nd	1.1	nd	1.7
1,2,3,4,6,7,8-HeptaCDF	0.7	1.4	0.7	1.9	0.9	1.0	1.2	19.8
1,2,3,4,7,8,9-HeptaCDF	nd	nd	nd	0.1	nd	nd	nd	2.1
OctaCDF	nd	nd	nd	0.3	nd	nd	0.6	1.3
TEQs (pg/g lipid)								
TEQ-PCDD/Fs	10.7	11.7	10.3	13.3	2.3	5.0	11.8	16.1

*n*: number of subjects; nd: not detected.

## Data Availability

The data presented in this study are available on request to the corresponding author.
